# The Impact of Magnet Recognition on Nurse Managers’ Assessments of Work Environment, Quality, and Safety: A Cross‐Sectional Study

**DOI:** 10.1155/jonm/1847111

**Published:** 2026-02-06

**Authors:** Hyunmin Yu, Luke J. Keele, Heather Brom, Helena Pittman, Roopa Varghese, Matthew D. McHugh, Linda H. Aiken

**Affiliations:** ^1^ Center for Health Outcomes and Policy Research, School of Nursing, University of Pennsylvania, Philadelphia, Pennsylvania, USA, upenn.edu; ^2^ Leonard Davis Institute of Health Economics, University of Pennsylvania, Philadelphia, Pennsylvania, USA, upenn.edu; ^3^ Department of Nursing, Penn Presbyterian Medical Center, Philadelphia, Pennsylvania, USA; ^4^ Department of Surgery, Perelman School of Medicine, University of Pennsylvania, Philadelphia, Pennsylvania, USA, upenn.edu; ^5^ Department of Regulatory Affairs, Temple Health, Philadelphia, Pennsylvania, USA

**Keywords:** Magnet hospitals, nurse managers, patient safety, quality of health care, work environment

## Abstract

**Background:**

Nurse managers are central to hospital operations, yet little is known about how organizational factors, such as Magnet Recognition by the American Nurses Credentialing Center (ANCC), shape their assessments of the work environment, care quality, and safety. This study examined whether Magnet status is associated with nurse managers’ assessments of these domains.

**Methods:**

This cross‐sectional study used data from the 2024 Penn Nurses4All Survey, the 2023 American Hospital Association Annual Survey, and the ANCC’s list of Magnet‐recognized organizations. The sample included 1362 nurse managers from 771 hospitals across 10 U.S. states (450 in 186 Magnet hospitals and 912 in 585 non‐Magnet hospitals). Outcomes included managers’ assessments of the work environment, nursing care quality, patient safety, and hospital recommendation. Approximate balancing weights were applied to adjust for hospital‐ and manager‐level covariates, and weighted linear probability models estimated average treatment effects.

**Results:**

Magnet managers were more likely to hold graduate degrees and to work in large, teaching, and not‐for‐profit hospitals. Compared with non‐Magnet managers, they were 9 percentage points more likely to rate the work environment as excellent or good (*b* = 0.09, 95% CI: 0.04–0.16) and to “definitely” recommend their hospital as a workplace (*b* = 0.09, 95% CI: 0.02–0.16); 7 percentage points more likely to rate nursing care quality as excellent or good (*b* = 0.07, 95% CI: 0.04–0.14); 12 percentage points more likely to assign an excellent or good patient safety grade (*b* = 0.12, 95% CI: 0.06–0.19); and 16 percentage points more likely to recommend their hospital to family and friends (*b* = 0.16, 95% CI: 0.08–0.24).

**Conclusion:**

Magnet status was associated with more favorable assessments of the work environment, quality, and safety among nurse managers. These findings suggest that Magnet structures may strengthen organizational environment and culture across multiple levels of the nursing workforce.

## 1. Introduction

As central leaders within healthcare organizations, nurse managers are responsible for a range of core operational and leadership functions, including stewardship of fiscal resources, supervision and development of nursing staff, and the implementation of complex organizational and policy initiatives. Through these responsibilities, they play a pivotal role in shaping hospital work environments and maintaining standards of care quality and safety [1]. Their roles are among the most demanding in the healthcare system. Shifting reimbursement models (e.g., pay‐for‐performance) and declining inpatient volumes have intensified executive pressures to increase productivity while improving quality and safety outcomes [2]. With the expansion of value‐based purchasing programs, nurse managers have become directly accountable for operational and clinical processes tied to reimbursement and quality metrics [3]. Because of these overlapping administrative and clinical demands, nurse managers’ perspectives on their hospital’s work environment, quality of care, and patient safety provide crucial insight into organizational performance and capacity for improvement.

Despite their central role in promoting safe, high‐quality care, relatively little research has examined how organizational factors shape nurse managers’ assessments of these domains. One organizational factor that remains underexplored is the Magnet Recognition Program (Magnet), administered by the American Nurses Credentialing Center (ANCC). Magnet is fundamentally designed to advance nursing excellence through structures and processes that support professional practice, leadership, and continuous quality improvement. Magnet designation signals an organizational commitment to creating environments in which nurses can deliver safe, high‐quality care while engaging in professional development and shared governance [4]. Prior research has linked Magnet hospitals to improved patient and workforce outcomes [5], including frontline nurses’ more favorable assessments of care quality and safety [6]. However, most evidence focuses on staff nurses and patient outcomes, with comparatively little attention to nurse managers, leaders whose perspectives directly influence the effectiveness of hospital systems. This study addresses this gap by examining whether Magnet status is associated with nurse managers’ assessments of the work environment, care quality, and patient safety.

### 1.1. Theoretical Framework

This study is guided by Donabedian’s model of healthcare quality [7], which conceptualizes quality through interrelated *structure*, *process*, and *outcome* domains. Structures encompass organizational characteristics of care delivery (e.g., resources, staffing, and governance). Processes capture the actions through which care is delivered (e.g., teamwork, communication, and leadership). Outcomes reflect the consequences of care (e.g., patient safety and quality of care). Strong structures enable effective processes, which in turn yield favorable outcomes [7].

Magnet represents a structural intervention that aligns with Donabedian’s model [7]. Hospitals recognized as Magnet institutions should demonstrate organizational excellence across five domains of the Magnet model: transformational leadership, structural empowerment, exemplary professional practice, new knowledge and innovation, and empirical outcomes [8]. These domains embody structural features that enable high‐quality processes. For example, Magnet’s emphasis on leadership and empowerment fosters environments where communication, collaboration, and decision‐making are more effective, which should translate into better outcomes for patients and the nursing workforce.

Although much of the Magnet literature centers on frontline nurses, Donabedian’s framework suggests that nurse managers, positioned at the nexus of organizational structures and frontline processes, are also likely to appraise Magnet‐related conditions favorably [9, 10]. Specifically, Magnet structures may translate into processes that enhance leadership effectiveness, bolster psychological safety, and support professional development. In turn, these processes are expected to yield more positive assessments of the work environment, quality of care, and patient safety. Accordingly, we hypothesize that nurse managers in Magnet hospitals will report more favorable evaluations of the work environment, care quality, and patient safety than their counterparts in non‐Magnet hospitals.

## 2. Materials and Methods

### 2.1. Study Design and Data Sources

This cross‐sectional observational study used three primary data sources: the 2024 Penn Nurses4All Study, the 2023 American Hospital Association (AHA) Annual Survey, and the list of Magnet‐recognized organizations. The Penn Nurses4All Study is a large‐scale survey in which nurses report on their work environments and employment conditions. The 2024 survey focused on nurse job outcomes and workplace settings in 10 U.S. states (CA, FL, IL, LA, NJ, NM, NY, OR, PA, and WA) [11–13]. In 2023 and 2024, actively licensed registered nurses in nine states were invited to participate by email using state licensure databases. In Pennsylvania, where only postal addresses were available, 30% of nurses were randomly selected to receive mailed invitations.

A modified Dillman method was applied, consistent with prior studies [14–16], and reminder notices were sent at regular intervals to nonrespondents [17]. Prior validation studies that used double‐sampling follow‐ups with nonrespondents found little evidence of nonresponse bias with this approach [14]. Information on hospital characteristics was obtained from the 2023 AHA Annual Survey, which provides data on hospital staffing, services, and operations [18]. Information on Magnet designation was drawn from the ANCC’s publicly available website [19].

### 2.2. Study Sample

**FIGURE 1:**
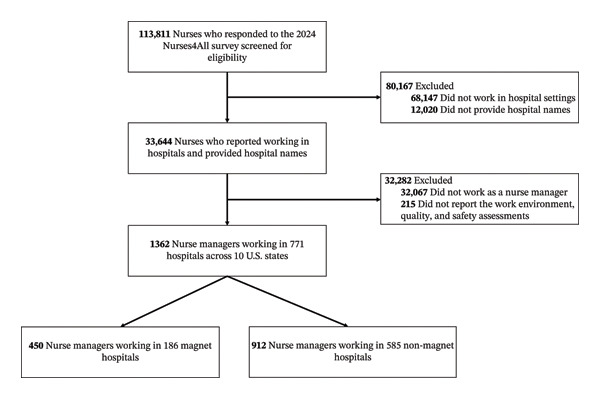
Sampling diagram. This figure depicts the process of selecting the analytic sample of nurse managers in hospital settings who participated in the 2024 Penn Nurses4All survey and reported on their assessments of the work environment, quality, and safety.

To examine the association between hospitals’ Magnet status and nurse managers’ assessments of the work environment, care quality, and patient safety, we included respondents who (1) identified their role as a nurse manager; (2) were employed in a hospital; (3) reported their hospital name; and (4) provided data on assessments of the work environment, quality, and safety. Of the 113,811 nurses who completed the 2024 Penn Nurses4All survey, those not meeting these criteria were excluded. The final analytic sample consisted of 1362 nurse managers from 771 hospitals across 10 U.S. states, including 450 nurse managers in 186 Magnet hospitals and 912 nurse managers in 585 non‐Magnet hospitals. The detailed sampling flow is shown in Figure 1.

### 2.3. Study Variables

#### 2.3.1. Dependent Variable I: Assessments of the Work Environment

Overall work environment was captured using the item, “How would you rate the overall work environment of your primary job?” Responses were provided on a 4‐point Likert scale: “1 = excellent,” “2 = good,” “3 = fair,” and “4 = poor.” For analysis, *excellent* and *good* were coded as 1, and *fair* and *poor* were coded as 0, distinguishing positive evaluations of the work environment from less positive ones.

Nurse managers’ likelihood of recommending their hospital as a workplace was assessed with the item, “Would you recommend your place of employment as a good place to work?” Responses used a 4‐point Likert scale: “1 = definitely yes,” “2 = probably yes,” “3 = probably not,” and “4 = definitely not.” For analysis, we dichotomized the item by coding *definitely yes* as 1 and all other options as 0, thereby distinguishing unequivocal endorsements from tentative or negative responses.

#### 2.3.2. Dependent Variable II: Assessments of Quality and Safety

The quality of nursing care was measured with a single‐item question that has shown strong associations with objective patient outcomes, including mortality [20, 21]. Participants were asked, “In general, how would you describe the quality of nursing care delivered to patients in your work setting?” Responses were given on a 4‐point Likert scale with options of “1 = excellent,” “2 = good,” “3 = fair,” and “4 = poor.” For analytic purposes, responses of *excellent* and *good* were recoded as 1, while *fair* and *poor* were coded as 0, differentiating favorable evaluations of care quality from less favorable ones.

Patient safety was evaluated using the single‐item question, “Please give your current practice setting an overall grade on patient safety.” This was answered on a 5‐point Likert scale: “1 = excellent,” “2 = good,” “3 = acceptable,” “4 = poor,” and “5 = failing.” For analysis, *excellent* and *good* were coded as 1, while *acceptable*, *poor*, and *failing* were coded as 0, thus separating strong positive perceptions of patient safety culture from neutral or negative assessments.

Nurse managers’ likelihood of recommending their hospital to family or friends was measured with the question, “Would you recommend where you work to your family and friends needing healthcare?” This measure has been validated internationally and has shown strong agreement between nurses’ and patients’ ratings of hospital care quality [22]. Responses were collected on a 4‐point Likert scale with options of “1 = definitely yes,” “2 = probably yes,” “3 = probably not,” and “4 = definitely not.” For analysis, *definitely yes* was coded as 1, and all other responses were coded as 0, allowing the measure to isolate strong, unequivocal endorsements of the hospital from more uncertain or negative responses.

#### 2.3.3. Independent Variable: Hospitals’ Magnet Status

The independent variable was a binary measure indicating whether the nurse manager worked in a Magnet hospital. This variable was coded as 1 for nurse managers employed at Magnet‐designated hospitals as of 2024, identified using the ANCC’s list of Magnet organizations [19], and coded as 0 for those employed in non‐Magnet hospitals.

#### 2.3.4. Nurse Manager‐Level Covariates

We included nurse manager‐level characteristics that have been associated with assessments of the work environment among nurse managers and staff nurses: age [23], gender [24], educational level [25], employment status [23], and work setting [26]. Age was treated as a continuous variable. Gender was recorded as *female* or *male*. Educational level (highest nursing degree) was categorized as *associate*, *baccalaureate*, *master’s*, or *doctoral*. Employment status was categorized as *full-time* or *part-time*. Work setting was categorized as *critical care unit*, *noncritical care unit*, or *nonpatient care (*e.g.*, administration or education)*.

#### 2.3.5. Hospital‐Level Covariates

We also included hospital‐level characteristics associated with the work environment, quality, and safety. These characteristics were hospital size, teaching status, specialized service capacity [27], and ownership [28, 29]. Teaching status was defined by the ratio of resident physicians and fellows to total hospital beds, a measure shown to be a more precise indicator of teaching intensity than university affiliation or membership in professional organizations [30]. Hospitals were classified as *nonteaching*, *minor teaching* (ratio ≤ 1:4), or *major teaching* (ratio > 1:4). Specialized service capacity was coded as *high* if the hospital offered advanced tertiary services such as open‐heart surgery or major organ transplantation [31]. Hospital size was categorized by number of beds as *small* (≤ 100), *medium* (101–250), or *large* (> 250) [27]. Ownership was categorized as *federal*, *for-profit*, or *not-for-profit*.

### 2.4. Statistical Analysis

All analyses were conducted using R (Version 4.5.1). We began with descriptive analyses of nurse manager‐ and hospital‐level characteristics. Next, we estimated the association between hospitals’ Magnet status and nurse managers’ assessments of the work environment, quality, and safety using a clustered observational study design [32, 33]. Because Magnet designation is assigned at the hospital (cluster) level, standard methods that assume independent treatment assignment at the individual level may produce biased results. To address this issue, we applied approximate balancing weights, a recently developed extension of inverse propensity weighting designed for clustered observational studies [34].

Balancing weights were obtained by solving a convex optimization problem that directly minimizes covariate imbalance between Magnet and non‐Magnet hospitals while imposing a variance penalty informed by intraclass correlation of nurse managers within hospitals. This approach allows weights to adjust simultaneously for both cluster‐level covariates (e.g., hospital size, ownership, teaching status, and specialized service capacity) and individual nurse manager‐level covariates (e.g., age, gender, degree, employment status, and work setting).

We estimated the average treatment effect for the treated (ATT), representing the expected difference in outcomes for nurse managers working in Magnet hospitals compared with what would have been observed had these same managers worked in non‐Magnet hospitals. Outcome models used a weighted linear probability model for binary outcomes (favorable ratings of quality, patient safety, work environment, and hospital recommendation). Standard errors and confidence intervals were estimated using cluster‐robust sandwich estimators that account for within‐hospital correlation. This variance estimation approach provides asymptotically valid inference under arbitrary within‐cluster dependence structures [34].

Among the 1362 nurse managers in the analytic sample, 300 (22.0%) had at least one missing value across the covariates, while 1062 had complete data. To address missingness, we imputed continuous covariates using the sample mean. For categorical covariates, we created a set of dummy variables for each category and balanced them separately, thereby ensuring that every category within a variable was represented in the balancing procedure. This approach preserves covariate balance, as imputing continuous variables with their mean does not alter the distribution of the observed data. In addition, for each covariate with missing values, we generated a binary indicator coded 1 if the value was missing and 0 otherwise. These indicators were included in the balance procedure, ensuring that not only the covariate distributions but also the patterns of missingness were balanced across treatment groups [35].

## 3. Results

Among the 1362 nurse managers, the mean age was 51.1 years (*SD* = 10.0), and the majority were female (86.8%) (Table 1). Most nurse managers worked full time (95.7%) and held a baccalaureate degree or higher (87.2%). By work setting, 61.2% were employed in noncritical care units, 20.3% in critical care units, and 18.5% in nonpatient care settings such as administration or education. Nurse managers in Magnet hospitals were more likely to hold a graduate degree compared with those in non‐Magnet hospitals (54.4% vs. 39.2%, *p* < 0.001).

**TABLE 1:** Nurse manager and hospital characteristics.

	**Nurse manager characteristics**			
	**Nurse managers in all hospitals (*N* = 1362)**	**Nurse managers in Magnet hospitals (*n* = 450)**	**Nurse managers in non-Magnet hospitals (*n* = 912)**	***p***

Age [(mean (*SD*)]	51.1 (10.0)	50.9 (10.4)	51.2 (9.8)	0.566
Highest degree [*n* (%)]^a^				< 0.001
Associate degree	160 (12.8)	18 (4.3)	142 (17.0)	
Baccalaureate degree	539 (43.0)	172 (41.3)	367 (43.8)	
Master’s degree	501 (40.0)	204 (49.1)	297 (35.5)	
Doctoral degree	53 (4.2)	22 (5.3)	31 (3.7)	
Gender [*n* (%)]^a^				0.979
Female	1064 (86.8)	353 (86.9)	711 (86.7)	
Male	162 (13.2)	53 (13.1)	109 (13.3)	
Employment status [*n* (%)]^a^				0.390
Full‐time	1300 (95.7)	434 (96.4)	866 (95.3)	
Part‐time	59 (4.3)	16 (3.6)	43 (4.7)	
Work setting [*n* (%)]^a^				0.586
Critical care unit	273 (20.3)	83 (18.8)	190 (21.0)	
Noncritical care unit	824 (61.2)	272 (61.7)	552 (61.0)	
Nonpatient care setting	249 (18.5)	86 (19.5)	163 (18.0)	

**Hospital characteristics**				
	**All hospitals (*N* = 771)**	**Magnet hospitals (*n* = 186)**	**Non-Magnet hospitals (*n* = 585)**	***p***

Hospital size [*n* (%)]^a^				< 0.001
Small (up to 100 beds)	149 (22.2)	2 (1.2)	147 (28.9)	
Medium (101‐250 beds)	204 (30.4)	34 (20.9)	170 (33.5)	
Large (over 250 beds)	318 (47.4)	127 (77.9)	191 (37.6)	
Teaching status [*n* (%)]^a^				< 0.001
Major	126 (18.8)	60 (36.8)	66 (13.0)	
Minor	309 (46.1)	52 (31.9)	257 (50.6)	
None	236 (35.1)	51 (31.3)	185 (36.4)	
Specialized service capacity [*n* (%)]^a^				< 0.001
High capacity	245 (36.5)	112 (68.7)	133 (26.2)	
Low capacity	426 (63.5)	51 (31.3)	375 (73.8)	
Ownership [*n* (%)]^a^				< 0.001
Not‐for‐profit	552 (82.3)	161 (98.8)	391 (77.0)	
For‐profit	92 (13.7)	1 (0.6)	91 (17.9)	
Federal	27 (4.0)	1 (0.6)	26 (5.1)	

Abbreviation: SD = standard deviation.

^a^The sum does not equal the total number because of missing data.

Across the 771 hospitals, most were not‐for‐profit (82.3%) and teaching hospitals (64.9%) (Table 1). Nearly half (47.4%) were large hospitals, and more than one‐third (36.5%) offered advanced services such as open‐heart surgery or major organ transplantation. Compared with non‐Magnet hospitals, Magnet hospitals were more likely to be large (77.9% vs. 37.6%, *p* < 0.001), teaching (68.7% vs. 63.6%, *p* < 0.001), have highly specialized service capacity (68.7% vs. 26.2%, *p* < 0.001), and be not‐for‐profit (98.8% vs. 77.0%, *p* < 0.001).

### 3.1. Unadjusted Outcome Differences

Table 2 presents unadjusted differences in outcomes between nurse managers in Magnet and non‐Magnet hospitals. Magnet hospitals showed consistently more favorable assessments of the work environment, quality, and safety. Nurse managers in Magnet hospitals were more likely to rate the work environment as excellent or good (74.7% vs. 63.3%, *p* < 0.001) and to “definitely” recommend their hospital as a good place to work (45.6% vs. 36.7%, *p* = 0.002). They were also more likely to evaluate nursing care positively (90.7% vs. 80.4%, *p* < 0.001) and to assign an excellent or good grade to patient safety (77.8% vs. 62.3%, *p* < 0.001). Finally, they were more likely to “definitely” recommend their hospital to family and friends needing healthcare (56.0% vs. 36.8%, *p* < 0.001).

**TABLE 2:** Unadjusted outcome differences among nurse managers by Magnet status.

	Nurse managers in all hospitals (*N* = 1362)	Nurse managers in Magnet hospitals (*n* = 450)	Nurse managers in non‐Magnet hospitals (*n* = 912)	*p*
Work environment assessments				
Overall work environment [*n* (%)]				< 0.001
Excellent or good	913 (67.0)	336 (74.7)	577 (63.3)	
Fair or poor	449 (33.0)	114 (25.3)	335 (36.7)	
Likelihood to recommend as a good place to work [*n* (%)]				0.002
Definitely yes	540 (39.6)	205 (45.6)	335 (36.7)	
Definitely no, probably no, or probably yes	822 (60.4)	245 (54.4)	577 (63.3)	
Quality and safety assessments				
Quality of nursing care [*n* (%)]				< 0.001
Excellent or good	1141 (83.8)	408 (90.7)	733 (80.4)	
Fair or poor	221 (16.2)	42 (9.3)	179 (19.6)	
Patient safety grade [*n* (%)]				< 0.001
Excellent or good	918 (67.4)	350 (77.8)	568 (62.3)	
Acceptable, poor, or failing	444 (32.6)	100 (22.2)	344 (37.7)	
Likelihood to recommend the hospital for family and friends needing care [*n* (%)]				< 0.001
Definitely yes	588 (43.2)	252 (56.0)	336 (36.8)	
Definitely no, probably no, or probably yes	774 (56.8)	198 (44.0)	576 (63.2)	

### 3.2. Effects of Magnet Status on Nurse Managers

Table 3 reports unweighted and weighted standardized mean differences (SMDs). After weighting, all absolute SMDs were less than 0.002, well below the commonly recommended 0.10 threshold, indicating excellent balance between groups. Table 4 presents the ATT estimates. Nurse managers in Magnet hospitals were 9% points more likely to rate their overall work environment as excellent or good (*b* = 0.09, 95% CI: 0.04–0.16) and 9% points more likely to “definitely” recommend their hospital as a good place to work (*b* = 0.09, 95% CI: 0.02–0.16) compared with those in non‐Magnet hospitals. They were also 7% points more likely to rate the quality of nursing care as excellent or good (*b* = 0.07, 95% CI: 0.04–0.14), 12% points more likely to assign an excellent or good patient safety grade (*b* = 0.12, 95% CI: 0.06–0.19), and 16% points more likely to recommend their hospital to family and friends needing healthcare (*b* = 0.16, 95% CI: 0.08–0.24).

**TABLE 3:** Unweighted and weighted standardized mean differences.

	Mean for non‐Magnet group	Mean for Magnet group	Unweighted standardized mean differences^a^	Weighted standardized mean differences
Age	51.184	50.853	−0.026	0.000
Gender^b^				
Female	0.780	0.784	0.010	0.000
Male	0.120	0.118	−0.004	0.000
Highest degree				
Associate degree	0.156	0.040	−0.358	−0.001
Baccalaureate degree	0.402	0.382	−0.034	0.000
Master’s degree	0.326	0.453	0.213	0.000
Doctoral degree	0.034	0.049	0.059	0.000
Employment status				
Full‐time	0.950	0.964	0.062	0.000
Part‐time	0.047	0.036	−0.049	0.000
Work setting				
Critical care unit	0.208	0.184	−0.049	0.000
Noncritical care unit	0.605	0.604	−0.001	0.000
Nonpatient care setting	0.179	0.191	0.026	0.000
Hospital size				
Small (up to 100 beds)	0.190	0.007	−0.633	0.000
Medium (101‐250 beds)	0.288	0.129	−0.344	0.000
Large (over 250 beds)	0.412	0.787	0.696	0.001
Teaching status				
Major	0.168	0.429	0.465	0.000
Minor	0.428	0.267	−0.285	0.000
None	0.295	0.227	−0.129	0.000
Specialized service capacity				
High capacity	0.304	0.698	0.700	0.000
Low capacity	0.587	0.224	−0.666	0.000
Ownership				
Not‐for‐profit	0.707	0.907	0.459	0.000
For‐profit	0.127	0.004	−0.501	0.000
Federal	0.056	0.011	−0.232	−0.001

^a^An absolute standardized mean difference above 0.1 is typically considered indicative of meaningful imbalance.

^b^For categorical variables, “Mean” represents the proportion in that category (e.g., 0.780 = 78.0% female). For multilevel variables (e.g., highest degree), each category was coded as a separate indicator (dummy) variable, and standardized mean differences were computed for each indicator. Row proportions may not sum to 1.00 because of rounding and because missing values were handled with a separate “missing” indicator included in the weighting.

**TABLE 4:** Average treatment effect for the treated (ATT) estimates.

	Estimate	SE	95% CI
Work environment assessments			
Overall work environment^a^	0.09	0.01	0.04–0.16
Likelihood to recommend as a good place to work	0.09	0.02	0.02–0.16
Quality and safety assessments			
Quality of nursing care	0.07	0.01	0.04–0.14
Patient safety grade	0.12	0.02	0.06–0.19
Likelihood to recommend the hospital for family and friends needing care	0.16	0.02	0.08–0.24

Abbreviations: ATT = average treatment effect for the treated, SE = standard error, and CI = confidence interval.

^a^The ATT estimate of 0.09 for “Overall work environment” means nurse managers in Magnet hospitals are 9% points more likely to rate their work environment as excellent or good than comparable managers in non‐Magnet hospitals.

## 4. Discussion

This study examined whether hospitals’ Magnet status is associated with nurse managers’ assessments of the work environment, care quality, and patient safety. We found that Magnet status was significantly associated with more favorable assessments of the work environment, quality of nursing care, patient safety, and the likelihood of recommending the hospital both as a workplace and for family and friends needing healthcare.

Magnet and non‐Magnet hospitals differ on factors that can independently influence managers’ assessments, such as hospital size, teaching status, ownership, and nurse manager characteristics, so simple unadjusted comparisons may make Magnet hospitals appear better (or worse) for reasons unrelated to Magnet status. Our weighting approach created a fairer comparison by giving greater emphasis to non‐Magnet managers who resembled Magnet managers on measured covariates and less emphasis to those who differed, while accounting for clustering. Overall, Magnet‐related structures may strengthen nurse managers’ assessments of the work environment, care quality, and patient safety.

Although some effects were modest (e.g., a 9‐percentage‐point increase in favorable work‐environment ratings), this likely reflects that the comparison group of non‐Magnet hospitals does not represent an absence of organizational commitment to quality, safety, or healthy work environments. Hospitals in this group may include former Magnet organizations, hospitals pursuing Magnet or other recognitions such as Pathway to Excellence [36], or institutions implementing similar quality‐focused initiatives independent of formal designation. This heterogeneity likely attenuates observed differences between groups and may partially explain the relatively narrow, yet statistically significant, effects observed in this study. Additionally, because we used clustered balancing weights to reduce confounding and cluster‐robust standard errors to account for within‐hospital correlation, our confidence intervals are conservative. As expected, adjusted point estimates are often smaller than naïve, unadjusted differences. Nonetheless, a 9% population‐level shift in binary outcomes can affect large numbers of units and patients, and even small, sustained gains in environment, safety, and quality have been linked to better patient outcomes [37].

### 4.1. The Ongoing Impact of Magnet Designation on Work Environment, Quality, and Safety

The alignment of our findings with prior evidence on frontline nurses reinforces the broader impact of Magnet designation as a structural intervention that shapes assessments of organizational quality and safety across multiple levels of the nursing workforce [38]. By promoting transformational leadership, structural empowerment, and professional practice standards, Magnet Recognition provides an organizational infrastructure that supports positive evaluations of care quality not only among bedside nurses but also among nurse managers, who play a critical role in bridging frontline practice and administrative leadership.

Magnet Recognition is fundamentally about advancing nursing excellence [39]. Nurse managers, who are themselves nurses, should be fully encompassed within the program’s vision. Extending Magnet efforts to intentionally support managerial practice environments (e.g., resourcing leadership development, ensuring manageable spans of control, formalizing mentorship, and embedding manager‐level recognition) could strengthen the durability of Magnet effects. Doing so would enhance the sustainability of Magnet hospitals by ensuring that all segments of the nursing workforce, clinical and managerial alike, experience the benefits of supportive organizational environments.

### 4.2. Limitations

This study has several limitations. First, while we partially mitigated confounding by applying approximate balancing weights for clustered observational data to align Magnet and non‐Magnet hospitals on observed hospital‐ and manager‐level covariates and to account for within‐hospital correlation, temporal ambiguity and unmeasured confounding may persist. Second, we excluded nurse managers who did not report work environment, quality, and safety assessments, which may have introduced selection bias. However, bivariate comparisons showed no statistically significant differences in demographics between respondents with complete data and those with missing data. Third, the sample was restricted to 10 U.S. states, which may limit generalizability. Despite these limitations, the study leverages a large, multistate sample of nurse managers and employs transparent weighting methods to improve comparability, offering practice‐relevant evidence on Magnet status and managerial assessments.

## 5. Conclusions

This study extends evidence on the Magnet Recognition Program by focusing on nurse managers, a critical yet understudied group. Magnet status was associated with more favorable assessments of the work environment, quality of care, patient safety, and likelihood of recommending the hospital. These findings suggest that Magnet structures may strengthen the organizational environment and the culture of quality and patient safety across multiple levels of the nursing workforce.

## Funding

This study was supported by the National Institute of Nursing Research, 10.13039/100000056, R01NR014855.

## Conflicts of Interest

Matthew D. McHugh reports receiving research funding from the National Institute of Nursing Research, National Institutes of Health (R01NR014855). Other authors declare no conflicts of interest.

## Data Availability

Research data are not shared.
